# LESI: A quantitative indicator to measure local environmental stewardship

**DOI:** 10.1016/j.mex.2020.101141

**Published:** 2020-11-12

**Authors:** John W. Turnbull, Emma L. Johnston, Graeme F. Clark

**Affiliations:** Biological, Earth and Environmental Science, University of NSW, Sydney, NSW, Australia

**Keywords:** Social-ecological systems, Conservation, Human behaviour

## Abstract

This method develops a local environmental stewardship indicator (LESI), which represents the level of stewardship action of a person at a place. The goal of the indicator is to quantify stewardship activity and allow it to be compared and modelled. LESI requires a brief interview to ascertain an individual's past and current stewardship activities, which are scored on a frequency scale for each of seven action categories. Scores are then combined using the LESI equation to:

• Quantify reported stewardship behaviour (as opposed to attitudes or intentions) as a single number.

• Enable comparisons of stewardship between individuals and places.

• Allow development of models to understand the predictors of stewardship, and

• Inform evidence-based strategies for stewardship improvement.

## Graphical abstract

**Figure:**
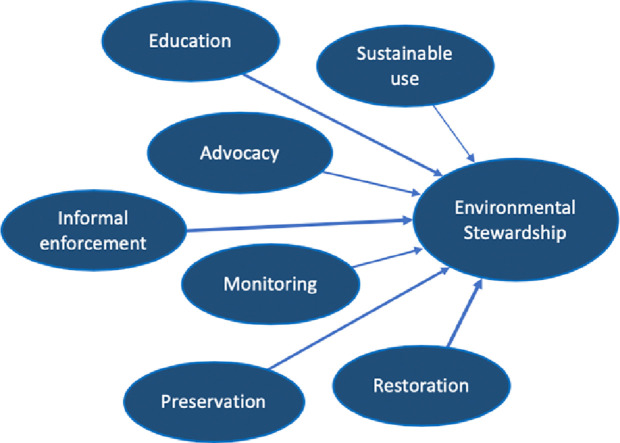

Specifications table

**Table:** 

Subject Area:	Earth and Planetary Sciences
More specific subject area:	Environmental conservation and stewardship, human behaviour
Method name:	LESI
Name and reference of original method:	Not applicable
Resource availability:	No specialist resources required

## Method details

### Background

With increasing calls for active earth stewardship [9,17], new concepts, frameworks and methods are required to ensure sustainable use and conservation of our natural resources and social and ecological values [5,^8^,22]. Recent studies have developed conceptual process models for environmental stewardship, such as Values-Tools-Outcomes [16,21], Origins-Practices-Outcomes [11] and Context-Actors-Motivations-Capacity-Actions-Outcomes [5].

Stewardship takes different forms; for example studies of civic and environmental stewardship in urban or agricultural settings [12,^16^,18]. It may involve social groups and organisations, often through volunteering (for example, [1]), however the understanding of environmental stewardship in less socially-structured contexts is less well developed [19].

Further research is required to elaborate and expand upon these concepts, explore social-ecological contexts, understand enablers in more depth and advance practical applications [5,7]. To date, little empirical research has targeted the relationship between the theory and practice of stewardship [3,7], particularly the development of social indicators, categories and predictive factors [4]. In this work, we develop a local environmental stewardship indicator (LESI) in order to quantify stewardship behaviour of individuals at a place. Our goal is to enable comparisons of stewardship levels, allow development of quantitative models to understand the predictors of stewardship, and ultimately inform evidence-based strategies for stewardship improvement.

### Development of LESI

Our research question was: can local environmental stewardship be quantified with an indicator across a broad social-ecological context?

We chose a coastal context in eastern Australia as our setting, as it encompassed urban and non-urban areas across terrestrial and marine realms. As stewardship is action-oriented, we focused on participant actions or behaviour. The Theory of Planned Behaviour [2] and cognitive hierarchy [20] theorise behaviour to be the result of behavioural intentions motivated by values, norms and attitudes. We focused our indicator on reported actions rather than intentions as the latter may be unrealised due to factors beyond a person's control, for example opportunity and resources [2]. Our focus on stewardship actions was in keeping with other recent studies, for example exploration of stewardship through the lens of social practice theory [13].

We initially recorded stewardship actions irrespective of classification in accordance with a grounded theory approach [10]. Grounded theory is one of the most widely applied analytical approaches in the study of qualitative data, with a suite of tools including theoretical sampling, emergent coding based on researcher interpretation, theoretical saturation and constant comparison to develop categories and the relationships between them [6]. Under this approach, themes and categories emerge over the duration of a study through rigorous analysis and inductive reasoning, grounded in the empirical evidence, with incorporation of ideas from existing literature being delayed until the final theoretical coding phase [6].

Our coding revealed seven categories of stewardship action, which we then used as input to principle components analysis (PCA). PCA revealed a single principal component with positive coefficients for all seven action categories, which forms the basis for the LESI equation.

### The LESI equation

Our work generated the following linear model:

LESI = 0.286*A_s_* + 0.384*A_e_* + 0.344*A_a_* + 0.46*A_n_* + 0.328*A_m_* + 0.377*A_p_* + 0.437*A_r_* where
LESI = Local environmental stewardship indicator for an individual at a place
*A_s_* = sustainable use
*A_e_* = education
*A_a_* = advocacy
*A_n_* = informal enforcement
*A_m_* = monitoring
*A_p_* = preservation
*A_r_* = restoration

Stewardship action categories and examples are provided in Table 1.

**Table 1:** The seven stewardship action categories revealed during coding, with examples of each from our coastal context.

Stewardship Action	Examples in the coastal context
Sustainable use	Using the natural resources of a site in a sustainable way, such as catch and release fishing, following sustainable fishing rules such as size and bag limits, sustainably collecting other resources such as kelp (wrack) and sea shells.
Education	Telling others about the site and its marine life (wildlife), explaining how to care for these (e.g. briefing people before diving), organizing and attending education events, and publishing information about local wildlife.
Advocacy	Advocating for a site and its marine life, such as signing a petition regarding stewardship of the site, writing to government, visiting and speaking with an official, attending rallies and participating in a “Friends of” group.
Informal enforcement	Acting to enforce conservation-related rules such as speaking to people breaking the rules, proactively explaining the rules to people, recording non-compliance or reporting it.
Monitoring	Keeping systematic records of the site including cataloguing and identifying photos and videos of wildlife, and participation in citizen science programs.
Preservation	Acting to prevent modification or development of the site, asking people not to fish at the site even though they are allowed, dune preservation and treading lightly (both above and under water).
Restoration	Acting to return the site to a prior state, such as picking up rubbish on land, collecting and disposing of rubbish on dives, participating in clean-up events above and under water, re-planting dunes, and watering plants.

Analysis of literature (part of the iterative coding process in grounded theory) confirmed that the above seven action categories provide comprehensive coverage of environmental stewardship in multiple contexts, beyond coastal (e.g. [15]). They also align with the four types of pro-environmental behaviour [14]; social environmentalism (education), land stewardship (preservation, monitoring), conservation lifestyle (restoration, sustainable use) and environmental citizenship (advocacy, informal enforcement).

### Application of LESI

The procedure for implementing LESI in a new context is as follows:

1. Develop study design including research questions and sampling strategy.
2. If data other than stewardship levels are to be collected, incorporate the LESI questions into the survey/interview instrument. To avoid biasing the response, ensure the LESI questions are asked before any questions that might lead to confirmation, compliance or social desirability bias.
3. For each participant, gain consent, then confirm the context, for example “The following questions relate to your activities at (define place)”
4. Ask the LESI question:

***Have you done anything, or do you regularly do anything, to help care for this place and its wildlife?***

5. In our experience, it is necessary to prompt for each of the seven categories in order to ensure complete coverage. We developed prompts based on Table 1, for example for the education category:

***Have you done anything to help educate others, such as tell them about this place and its wildlife, and how to look after it?***
Prompts and examples can be refined to suit the particular context of the study.

6. Seek confirmation from participants to allow accurate scoring, for example if a participant says “yes” ask:

***Can you give me some recent examples / can you tell me more about that?***

7. Score each participant for each of the seven categories, using the 0-4 scale:

  0 = action never taken at site,
  1 = action taken once, often as an exception to regular behaviour,
  2 = action taken sometimes, often incidentally to other priorities,
  3 = action taken most times the participant was at site, and
  4 = action taken always when possible on site, as a matter of priority.

8. On completion of data collection, conduct principal component analysis (PCA) to determine the weightings for the context of the study. PCA may resolve one or more principal components, with the coefficients of the first principal component representing the LESI weightings.
9. Calculate the stewardship score for each participant using the LESI equation, using the weightings in step (8).
10. To determine site-level stewardship, calculate average or maximum stewardship across all participants at the site. We found maximum stewardship to be a good indicator as it represents the activities of the most active stewards at a site – “uber-stewards” [19] – that may have the most impact on the site, for example through regular clean-ups, advocacy or establishment of norms.

### Validation

The above method resulted in an indicator which represented 36.5% of variance in our multivariate model [19]. We found our stewardship indicator to be well aligned with our qualitative data set, confirmed through several iterations of coding with an inter-coder reliability of 96.5%. The resulting LESI scores were sufficiently resolved to achieve significant results (*p*<0.05) in our univariate mixed method model, for four predictors of stewardship [19].

## Declaration of Competing Interest

The Authors confirm that there are no conflicts of interest.
